# Tetraphenylethylene-conjugated polycation covered iron oxide nanoparticles for magnetic resonance/optical dual-mode imaging

**DOI:** 10.1093/rb/rbab023

**Published:** 2021-06-14

**Authors:** Li Yang, Shengxiang Fu, Li Liu, Zhongyuan Cai, Chunchao Xia, Bin Song, Qiyong Gong, Zhiyun Lu, Hua Ai

**Affiliations:** 1 National Engineering Research Center for Biomaterials, Sichuan University, Chengdu 610065, China; 2 Department of Radiology, West China Hospital, Sichuan University, Chengdu 610041, China; 3 Huaxi MR Research Center (HMRRC), Department of Radiology, West China Hospital of Sichuan University, Chengdu, China; 4 Psychoradiology Research Unit of Chinese Academy of Medical Sciences, Sichuan University, Chengdu, China; 5 Key Laboratory of Green Chemistry and Technology (Ministry of Education), College of Chemistry, Sichuan University, Chengdu 610065, China

**Keywords:** magnetic resonance imaging, optical imaging, superparamagnetic iron oxide, aggregation-induced emission, dual-mode imaging

## Abstract

Magnetic resonance (MR)/optical dual-mode imaging with high sensitivity and high tissue resolution have attracted many attentions in biomedical applications. To avert aggregation-caused quenching of conventional fluorescence chromophores, an aggregation-induced emission molecule tetraphenylethylene (TPE)-conjugated amphiphilic polyethylenimine (PEI) covered superparamagnetic iron oxide (Alkyl-PEI-LAC-TPE/SPIO nanocomposites) was prepared as an MR/optical dual-mode probe. Alkyl-PEI-LAC-TPE/SPIO nanocomposites exhibited good fluorescence property and presented higher *T*_2_ relaxivity (352 Fe mM^−1^s^−1^) than a commercial contrast agent Feridex (120 Fe mM^−1^s^−1^) at 1.5 T. The alkylation degree of Alkyl-PEI-LAC-TPE effects the restriction of intramolecular rotation process of TPE. Reducing alkane chain grafting ratio aggravated the stack of TPE, increasing the fluorescence lifetime of Alkyl-PEI-LAC-TPE/SPIO nanocomposites. Alkyl-PEI-LAC-TPE/SPIO nanocomposites can effectively labelled HeLa cells and resulted in high fluorescence intensity and excellent MR imaging sensitivity. As an MR/optical imaging probe, Alkyl-PEI-LAC-TPE/SPIO nanocomposites may be used in biomedical imaging for certain applications.

## Introduction

Recently, aggregation-induced emission (AIE) [1, 2] has been widely studied and used in biomedical imaging applications, such as non-specific cell imaging [3], specific organelle imaging [4] and long-term cell tracking [5]. Different from conventional fluorescence chromophores’ aggregation-caused quenching, AIE molecules have significantly enhanced fluorescence in the aggregated state [1, 2, 6]. AIE nanoprobes with excellent photostability and easy cell internalization have attracted attentions in biomedical imaging, but weak tissue penetration and low spatial resolution limit their further applications [7, 8].

Multi-mode imaging modalities such as magnetic resonance (MR)/optical [9, 10], MR/positron emission tomography (PET) [11, 12] and PET/computed tomography [13, 14] with complementary information about anatomical structure and function have been extensively explored and developed in biomedical applications. MR/optical imaging has its unique advantages. MRI is a non-invasive medical imaging technique with high spatial resolution to acquire subtle anatomical and functional information of tissues and organs. However, the sensitivity of MRI is relatively low than other imaging modalities such as PET or optical imaging. The combination of these MRI and optical imaging modalities enables one to achieve both detailed tissue resolution and high sensitivity. Recently, the clinically used gadolinium (Gd)-based MR *T_1_* contrast agents have been reported to combine with AIE molecules as a dual-mode imaging probe which realized the liver-specific imaging [15], the visualization and quantification of changes in brain barrier functions [16]. However, Gd-based MR contrast agents have a strong correlation with nephrogenic systemic fibrosis in patients with renal insufficiency, which limits their applications [17, 18]. Due to their excellent biosafety, high sensitivity and easy modification, iron oxide nanoparticles (IONPs)-based MR *T*_2_ contrast agents have been used for cancer diagnosis [19, 20], atherosclerosis diagnosis [21, 22], long-term cell tracing [23, 24] in clinical trials. In addition, previous studies showed that multiple superparamagnetic iron oxide (SPIO) nanocrystal containing micelles have much higher *T*_2_ relaxivity than single SPIO nanocrystal suspensions at the same Fe concentration [25]. The controlled aggregation of SPIO nanocrystals can enhance relaxivity, similar to the effects that AIE molecules brought to us. So, the development of MR/optical imaging probes based on IONP and AIE molecules may find its unique applications.

**Scheme 1.**
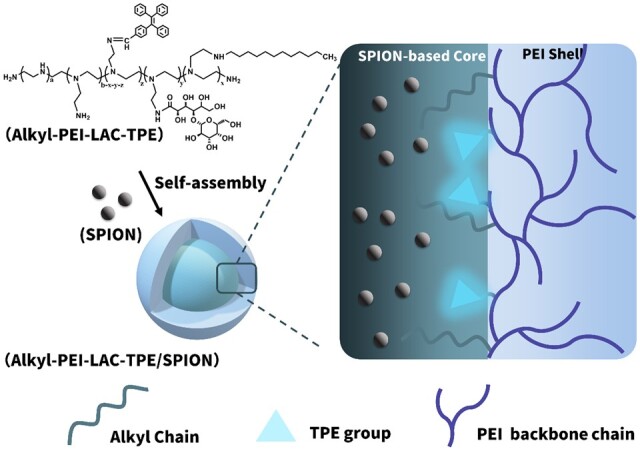
The illustrated structure of Alkyl-PEI-LAC-TPE/SPIO nanocomposites

Herein, an MR/optical imaging probe based on Alkyl-PEI-LAC-TPE/SPIO nanocomposites was developed (Scheme 1). Amphiphilic polymer Alkyl-PEI-LAC-TPE was designed for encapsulation of hydrophobic SPIO nanocrystals, the modification of lactobionic acid (LAC) on alkyl-polyethylenimine (PEI) was used to reduce the toxicity of PEI and the conjugation of tetraphenylethylene (TPE) endowed both hydrophobic and AIE features. Besides, different grafting degrees of alkane chain (C_12_) on polymer were used to understand the impact of amphiphilic structure on TPE’s fluorescence property. Then, SPIO nanocrystals were encapsulated inside the hydrophobic core of Alkyl-PEI-LAC-TPE micelles by self-assembly. The size distribution and morphology of Alkyl-PEI-LAC-TPE/SPIO nanocomposites were investigated, and the fluorescence properties and *in vitro* fluorescence imaging were evaluated, respectively. Additionally, *T*_2_ relaxivity and *in vitro* MRI study of Alkyl-PEI-LAC-TPE/SPIO nanocomposites were tested under clinical MRI scanners.

## Materials and methods

### Materials

PEI (MW:1.8 kD), N-Hydroxysuccinimide (NHS, 98%), N-(3-Dimethylaminopropyl)-N′-ethylcarbodiimide hydrochloride (EDC, 98%) and 1-iodododecane (>98%) were purchased from Aladdin Industrial Corporation. Aldehyde functionalized tetraphenylethene (TPE-CHO) was purchased from AIEgen Biotech Co., Ltd. Lactobionic acid (LAC) was purchased from TCI (Shanghai) Chemical Industry Development Corporation. Iron (III) acetylacetonate (97%), 1,2-hexadecanediol (90%), benzyl ether (98%), oleic acid (70%) and oleylamine (70%) were purchased from Sigma-Aldrich Corporation. All other chemicals and solvents were of analytical grade and used without further purification.

### Synthesis of Alkyl-PEI-LAC-TPE polymer

The synthesis route of Alkyl-PEI-LAC-TPE was displayed in Fig. S1 (online supplementary material). PEI-LAC was synthesized followed the reported protocol [26] with slight modification. Then, TPE-CHO was added for reaction with the amine of PEI-LAC. 1-Iodododecane was reacted with PEI-LAC-TPE in dimethyl sulfoxide (DMSO)/dimethylformamide (DMF) mixed solution following a previously reported method [25, 27] with slight modification.

PEI (4.3 g, 100 mmol) was dissolved in absolute DMSO (30 ml). LAC (3.58 g, 10 mmol), EDC (2.1 g, 11 mmol) and NHS (1.27 g, 11 mmol) were added and dissolved in anhydrous DMSO (40 ml) in a 100-ml round-bottom flask, and performed at 30°C for 4 h under stirring. The activated LAC was added dropwise to a stirring solution of PEI under nitrogen. The reaction was performed at 30°C for 24 h under stirring. The crude product (PEI-LAC) was dialyzed against deionized water (molecular mass cutoff, 1 kDa), collected and freeze-dried.

PEI-LAC (5.5 g, 70 mmol) and TPE-CHO (2.5 g, 7 mmol) were dissolved in anhydrous DMSO, respectively. TPE-CHO was added dropwise to a stirring solution of PEI-LAC, and the reaction was performed at 55°C for 18 h under stirring. The crude product was added dropwise to cold ethyl ether, and the target product PEI-LAC-TPE in the bottom was collected, dialyzed against deionized water and freeze-dried.

1-Iodododecane (0.24 g, 0.8 mmol) was added dropwise to PEI-LAC-TPE (0.92 g, 8 mmol) in DMSO/DMF mixed solution under nitrogen, and heated to 55°C for 6 h with stirring. After cooling to room temperature, the solution was incubated overnight. The crude product was dialyzed against gradient EtOH/H_2_O solution. By controlling the ratio of 1-iodododecane and PEI-LAC-TPE, Alkyl-PEI-LAC-TPE with different alkane grafting degrees were synthesized. The crude product (Alkyl-PEI-LAC-TPE) was collected and freeze-dried. Alkyl-PEI-LAC-TPE with different grafting degrees of alkane chains were synthesized to investigate probes’ fluorescence properties.

### Characterization of Alkyl-PEI-LAC-TPE polymer

^1^H NMR spectra of PEI, PEI-LAC, PEI-LAC-TPE and Alkyl-PEI-LAC-TPE were acquired on an NMR spectrometer (Bruker, 400 MHz). Elemental analysis of PEI, PEI-LAC and PEI-LAC-TPE were acquired on an element analyser (Teledyne Leeman Labs). Fourier-transform infrared spectroscopy (FTIR) spectra of PEI, PEI-LAC, PEI-LAC-TPE and Alkyl-PEI-LAC-TPE were recorded on an FTIR spectrometer (Thermo Fisher Scientific).

### Synthesis of SPIO nanocrystals

SPIO nanocrystals were synthesized following a reported method from Sun *et al.* [28]. Briefly, Fe(acac)_3_ (2 mmol), 1,2-hexadecanediol (2 mmol), oleic acid (2 mmol) and oleylamine (2 mmol) were added in benzyl ether (20 ml) with nitrogen. The mixture was heated to 200°C for 2 h, then the mixture was heated to reflux for 1 h. After cooling to room temperature, ethyl acetate was used to yield a precipitate from the solution. Then, the product was dispersed in hexane.

### Preparation of Alkyl-PEI-LAC-TPE micelles and SPIO-loaded Alkyl-PEI-LAC-TPE (Alkyl-PEI-LAC-TPE/SPIO) nanocomposites

SPIO nanocrystals in hexane were dried with nitrogen, then redispersed in chloroform. Alkyl-PEI-LAC-TPE was dissolved in DMSO, then added into chloroform under sonication. Then, the mixture of Alkyl-PEI-LAC-TPE with SPIO at a mass ratio of 3:1 was added into deionized water under sonication and kept shaking for 0.5 h. Chloroform was removed from by evaporation and dialyzed against deionized water to clear DMSO to obtain Alkyl-PEI-LAC-TPE/SPIO nanocomposites. The fabrication of Alkyl-PEI-LAC-TPE micelles was following the same protocol.

### Size distribution and morphology of SPIO nanocrystals, Alkyl-PEI-LAC-TPE micelles and Alkyl-PEI-LAC-TPE/SPIO nanocomposites

The size distribution of SPIO, Alkyl-PEI-LAC-TPE and Alkyl-PEI-LAC-TPE/SPIO nanocomposites was performed at 25°C via dynamic light scattering (DLS) (Zetasizer Nano ZS, Malvern Instruments). The surface morphology of Alkyl-PEI-LAC-TPE micelles and Alkyl-PEI-LAC-TPE/SPIO nanocomposites were investigated by scanning electron microscopy (S-4800, Hitachi), for which 50 µl of the samples were dried on a piece of silicon. The size distribution and ultrastructure of Alkyl-PEI-LAC-TPE micelles and Alkyl-PEI-LAC-TPE/SPIO nanocomposites were studied by transmission electron microscopy (TEM) (Tecnai G2 F20 S-TWIN, FEI), for which 10 µl of the samples were dried on a copper grid.

### Fluorescence properties of Alkyl-PEI-LAC-TPE micelles and Alkyl-PEI-LAC-TPE/SPIO nanocomposites

The ultraviolet absorption (UV) spectrum of Alkyl-PEI-LAC-TPE micelles was obtained by ultraviolet–visible (UV–VIS) spectrophotometer (U-3900, Hitachi). Then, the excitation spectrum, emission spectrum and fluorescence intensity at the maximum emission wavelength of Alkyl-PEI-LAC-TPE micelles were recorded using fluorescence spectrophotometer (F-7000, Hitachi) with a slit width of 5.0 nm. What is more, the fluorescence lifetime of Alkyl-PEI-LAC-TPE and Alkyl-PEI-LAC-TPE/SPIO nanocomposites were investigated by the transient fluorescence spectrophotometer (TEMPRO-01, Horiba).

### *T*_2_ relaxivity of Alkyl-PEI-LAC-TPE/SPIO nanocomposites

The *T*_2_ relaxivity of Alkyl-PEI-LAC-TPE/SPIO was measured on a 1.5 T clinical MRI scanner (Siemens): Repetition time (TR) = 5000 ms, Spin echo (TE) values ranging from 6.9 to 500 ms. Samples were prepared with series Fe concentrations (0.03, 0.06, 0.1, 0.15, 0.25, 0.3, 0.4 and 0.5 mM) of Alkyl-PEI-LAC-TPE/SPIO, and Fe concentrations of Alkyl-PEI-LAC-TPE/SPIO nanocomposites were measured by atomic absorption spectrum.

### *In vitro* fluorescence imaging of labelled HeLa cells with Alkyl-PEI-LAC-TPE micelles and Alkyl-PEI-LAC-TPE/SPIO nanocomposites

HeLa cells were cultured in DMEM medium containing 10% FBS at 37°C with 5% CO_2_. HeLa cells were plated at a density of 5 × 10^4^ cell per well in a 24-well plate. After cell attachment, Alkyl-PEI-LAC-TPE micelles and Alkyl-PEI-LAC-TPE/SPIO nanocomposites were diluted into DMEM medium with TPE concentration of 2.5 µg/ml for 12 h. Next, the medium was removed and the cells were washed with PBS twice. After the cells were fixed with 4% paraformaldehyde for 15 min, the fluorescence images were captured under a confocal laser scanning microscope (CLSM). The mean fluorescence intensities of HeLa cells incubated with Alkyl-PEI-LAC-TPE micelles and Alkyl-PEI-LAC-TPE/SPIO nanocomposites were calculated by Image J.

### *In vitro* MRI study of the labelled HeLa cells with Alkyl-PEI-LAC-TPE/SPIO nanocomposites

HeLa cells were labelled with different Fe concentrations of Alkyl-PEI-LAC-TPE/SPIO, and the intracellular iron content was detected by colorimetric ferrozine assay to detect the label efficiency of Alkyl-PEI-LAC-TPE/SPIO. Besides, *in vitro* MRI study of the labelled HeLa cells with Alkyl-PEI-LAC-TPE/SPIO nanocomposites were measured on a 3.0 T clinical MRI scanner (Siemens): TR = 5000 ms, TE values ranging from 6.9 to 500 ms. Briefly, HeLa cells were incubated with Alkyl-PEI-LAC-TPE/SPIO nanocomposites for 12 h at an Fe concentration of 5 µg/ml, respectively. Cells were collected and washed, then fixed with 4% paraformaldehyde for 30 min. Labelled cells were mixed with 5% gelatine crosslinked with glutaraldehyde in 250 µl microcentrifuge tubes in different cell numbers (0.5 × 10^4^, 1 × 10^5^, 2.5 × 10^5^ and 5 × 10^5^), and unlabelled cells were used as blank control. MR imaging was performed and *T*_2_ relaxation time was detected.

## Results and discussion

### Synthesis and characterization of Alkyl-PEI-LAC-TPE polymer

**Figure 1.**
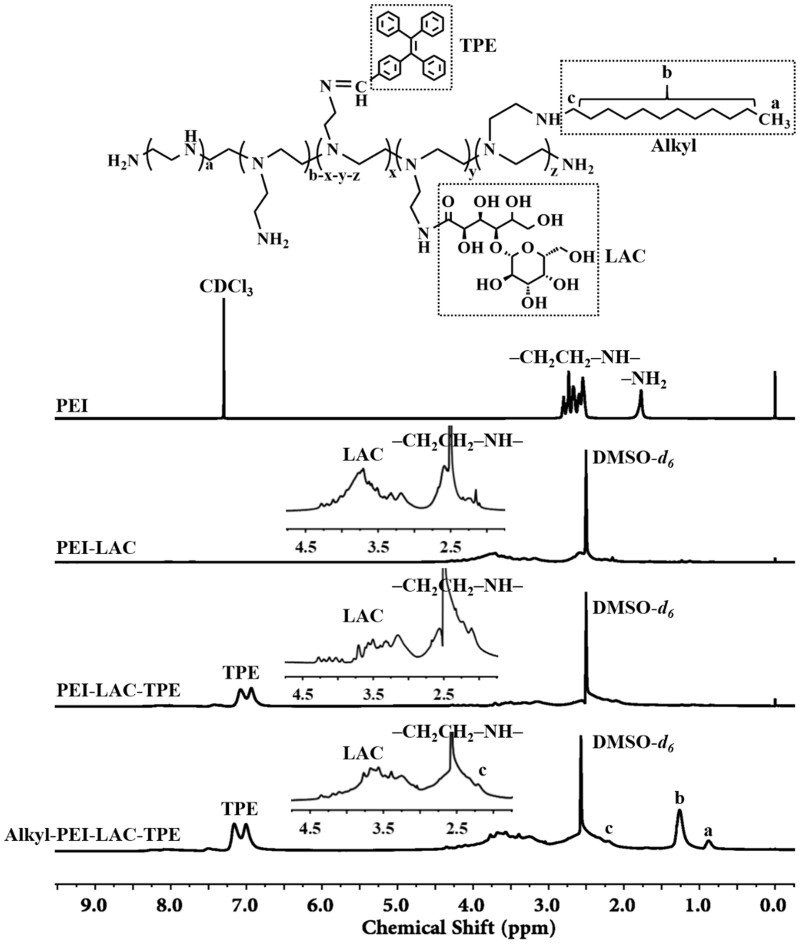
^1^H NMR spectrum of PEI, PEI-LAC, PEI-LAC-TPE and Alkyl-PEI-LAC-TPE

The ^1^H NMR spectrum of PEI, PEI-LAC, PEI-LAC-TPE and Alkyl-PEI-LAC-TPE was shown in Fig. 1. Low molecular weight branched PEI was firstly modified with LAC to improve its biocompatibility. Characteristic peaks of PEI-LAC were appeared in the ^1^H NMR spectrum (400 MHz, DMSO-*d_6_*): δ (ppm) 4.33–2.96 (**LAC**), 2.81–2.09 (–**CH_2_**–**CH_2_**–NH–). TPE with good AIE effect was then conjugated with PEI-LAC. Characteristic peaks of PEI-LAC-TPE were appeared in the ^1^H NMR spectrum (400 MHz, DMSO-*d_6_*): δ (ppm) 7.19–6.74 (**TPE**), 4.39–2.91 (**LAC**), 2.83–1.90 (–**CH_2_**–**CH_2_**–NH–). To explore the effect of alkane chains on the fluorescence properties of TPE, PEI-LAC-TPE was modified with different degrees of alkane chains by reacting with 1-iodododecane. Characteristic peaks of Alkyl-PEI-LAC-TPE were appeared in the ^1^H NMR spectrum (400 MHz, DMSO-*d_6_*): δ (ppm) 7.22–6.74 (**TPE**), 4.31–2.94 (**LAC**), 2.89–1.90 [–**CH_2_**–**CH_2_**–NH–, –**CH_2_**–(CH_2_)_10_–CH_3_], 1.31–1.02 [–CH_2_–**(CH_2_)_10_**–CH_3_], 0.81 [–CH_2_–(CH_2_)_10_–**CH_3_**]. The ^1^H NMR spectrum of Alkyl-PEI-LAC-TPE with different grafting degrees of alkane chains was displayed in Fig. S2 (online supplementary material).

**Figure 2.**
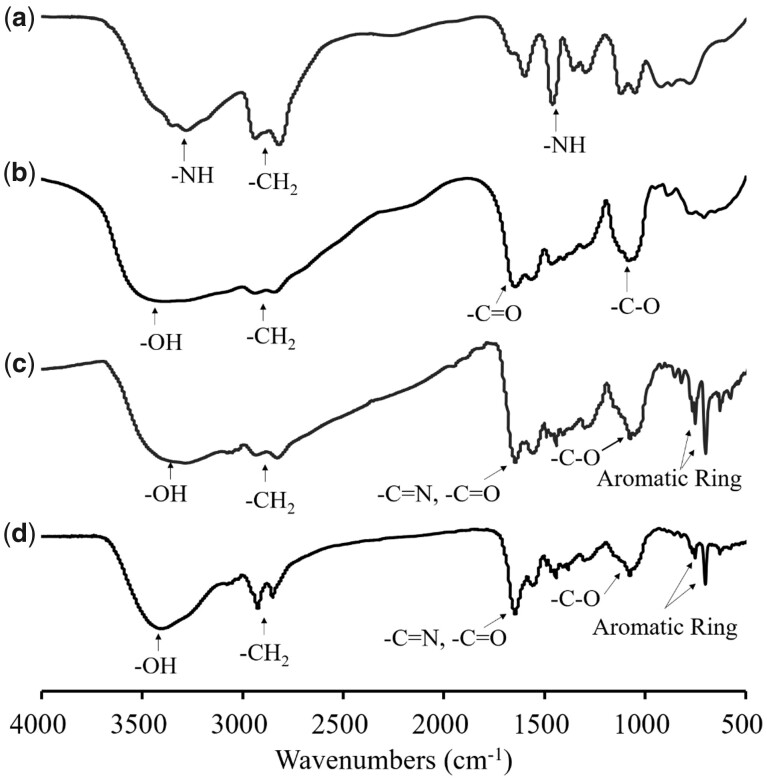
FTIR spectra of PEI (**a**), PEI-LAC (**b**), PEI-LAC-TPE (**c**) and Alkyl-PEI-LAC-TPE (**d**)

FTIR was used to characterize PEI-LAC, PEI-LAC-TPE and Alkyl-PEI-LAC-TPE. As shown in Fig. 2, the characteristic peaks at 3290 and 1460 cm^−1^ were attributed to the -NH and at 2940–2820 cm^−1^ were attributed to the -CH_2_ of PEI. The characteristic peaks at 3410 cm^−1^ (-OH), 1650–1560 cm^−1^ (-C = O) and 1060 cm^−1^ (-C-O) were attributed to LAC, indicating the successful synthesis of PEI-LAC. For PEI-LAC-TPE, a peak of the stretching vibration of aromatic rings of TPE at 750–700 cm^−1^ could be seen. The -CH_2_ peak of Alkyl-PEI-LAC-TPE at 2940–2820 cm^−1^ was strengthened after modified with alkane chains.

The LAC and TPE grafting ratios were calculated by elemental analysis according to the changes of ratio of carbon (C) and nitrogen (N) in PEI, PEI-LAC and PEI-LAC-TPE. The results showed that the LAC and TPE grafting ratios were 11% and 10%, respectively. Besides, the alkane chain grafting ratio was calculated by the ^1^H NMR of Alkyl-PEI-LAC-TPE according to the integral values at δ 7.22–6.74 and δ 0.81 ppm (Fig. 1 and Fig. S2, online supplementary material). Results showed that the alkane chain grafting ratios of Alkyl-PEI-LAC-TPE were 13%, 27% and 40%, respectively.

### Size distribution and morphology of SPIO nanocrystals, Alkyl-PEI-LAC-TPE micelles and Alkyl-PEI-LAC-TPE/SPIO nanocomposites

**Figure 3.**
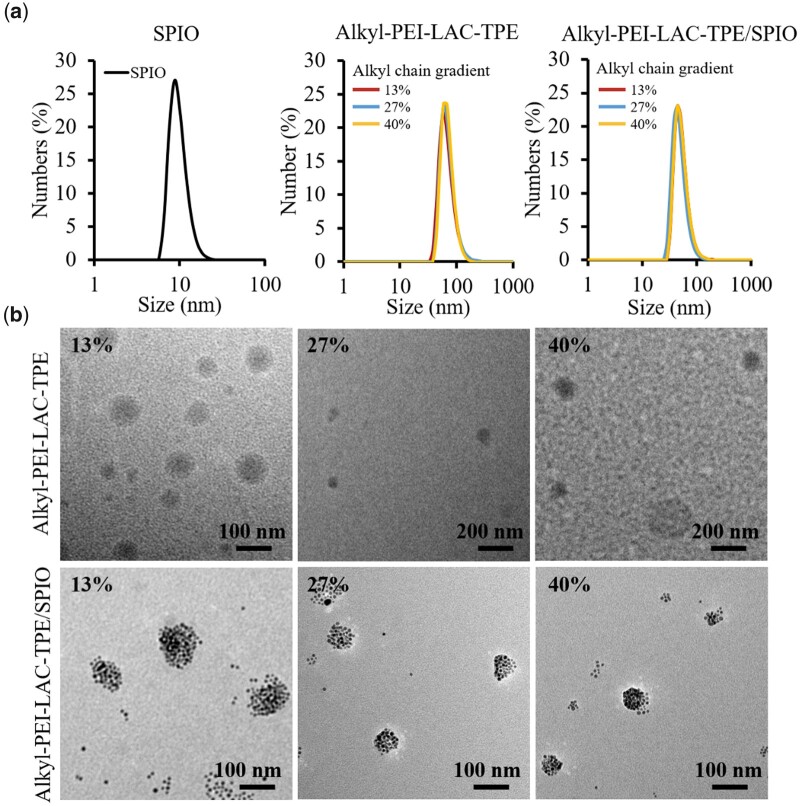
(**a**) DLS of SPIO nanocrystals in hexane, Alkyl-PEI-LAC-TPE micelles and Alkyl-PEI-LAC-TPE/SPIO nanocomposites in H_2_O. (**b**) TEM images of Alkyl-PEI-LAC-TPE micelles and Alkyl-PEI-LAC-TPE/SPIO nanocomposites with indicated alkyl chain gradients

Organic SPIO nanocrystal has a narrow size distribution with a mean diameter of 9 nm characterized by DLS in n-hexane (Fig. 3). Alkyl-PEI-LAC-TPE and Alkyl-PEI-LAC-TPE/SPIO nanocomposites with gradient alkane chains were prepared by self-assembly in deionized water. Alkyl-PEI-LAC-TPE micelles with gradient alkane chains have a similar diameter of 70 nm measured by DLS (Fig. 3), indicating that the different of alkane chain grafting ratios did not have significant impact on their size distribution. Alkyl-PEI-LAC-TPE/SPIO nanocomposites with gradient alkyl chains have a similar diameter of 50 nm in DLS, smaller than that of Alkyl-PEI-LAC-TPE micelles. TEM results (Fig. 3) showed that Alkyl-PEI-LAC-TPE micelles were spherical-like structure, and organic SPIO nanocrystals were encapsulated in Alkyl-PEI-LAC-TPE micelles as isolated spherical nanocluster structure.

### Fluorescence properties of Alkyl-PEI-LAC-TPE micelles and Alkyl-PEI-LAC-TPE/SPIO nanocomposites

**Figure 4.**
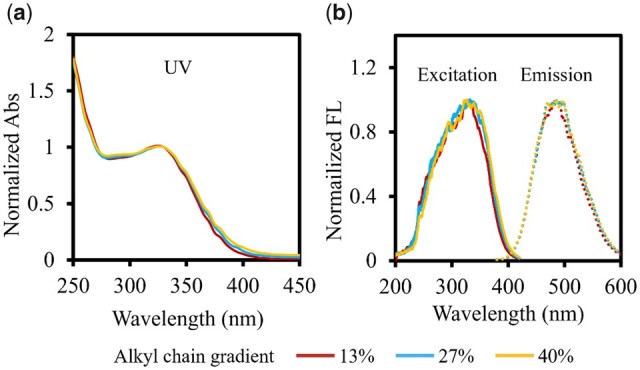
UV spectrum (**a**), excitation spectrum and emission spectrum (**b**) of gradient alkyl chain Alkyl-PEI-LAC-TPE micelles in H_2_O

As fluorescence probes, the fluorescence properties of Alkyl-PEI-LAC-TPE micelles and Alkyl-PEI-LAC-TPE/SPIO nanocomposites were studied, respectively. As shown in Fig. 4, the UV, excitation and emission spectrum of Alkyl-PEI-LAC-TPE micelles of different alkyl chains were the same in deionized water, with max excitation wavelength of 330 nm and max emission wavelength of 480 nm, indicating that Alkyl-PEI-LAC-TPE micelles with gradient alkyl chains had same fluorescent chromophore. Solid powders of gradient alkyl chain Alkyl-PEI-LAC-TPE appeared yellow at day-light and emitted a blue light excited by a UV-light (Fig. S3, online supplementary material).

**Figure 5.**
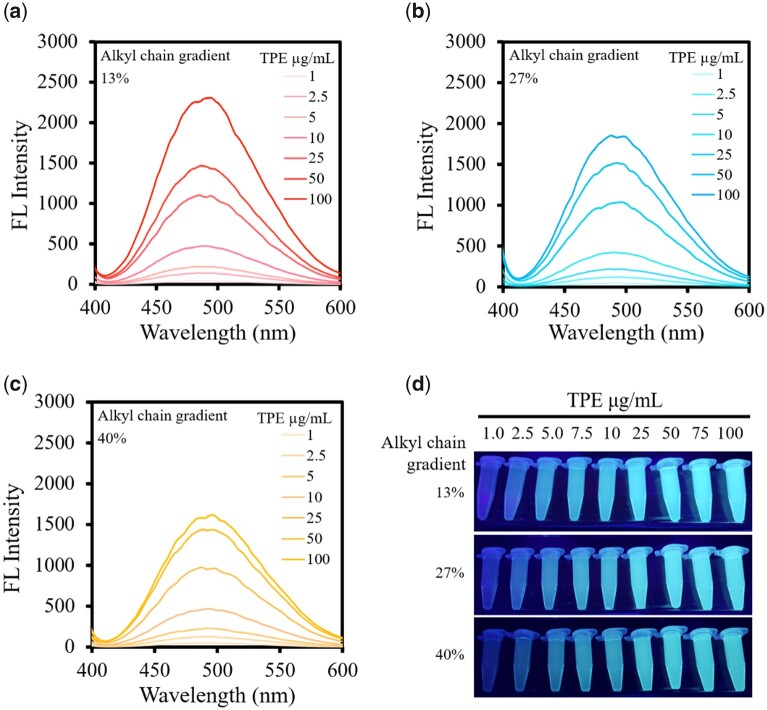
The fluorescence intensity (**a–c**) and the fluorescence images (**d**) of gradient alkyl chain Alkyl-PEI-LAC-TPE micelles at a series of TPE concentrations

The fluorescence intensity of gradient alkyl chain Alkyl-PEI-LAC-TPE micelles was measured at a series of TPE concentrations to evaluate the effect of alkane chain grafting ratios on the fluorescence properties of Alkyl-PEI-LAC-TPE. As shown in Fig. 5, the fluorescence intensity of Alkyl-PEI-LAC-TPE micelles with different alkyl chains was increased with the increasing of TPE concentration. For the same TPE concentration, the fluorescence intensity of Alkyl-PEI-LAC-TPE micelles was ranging from strong to weak with 13%, 27% and 40% alkyl chains (Fig. 5). In other words, the lower of alkane chain grafting ratio, the higher of fluorescence intensity at the same TPE concentration. It was possible that the different degrees of alkane chain grafting ratios of Alkyl-PEI-LAC-TPE had an effect on the fluorescence property.

**Figure 6.**
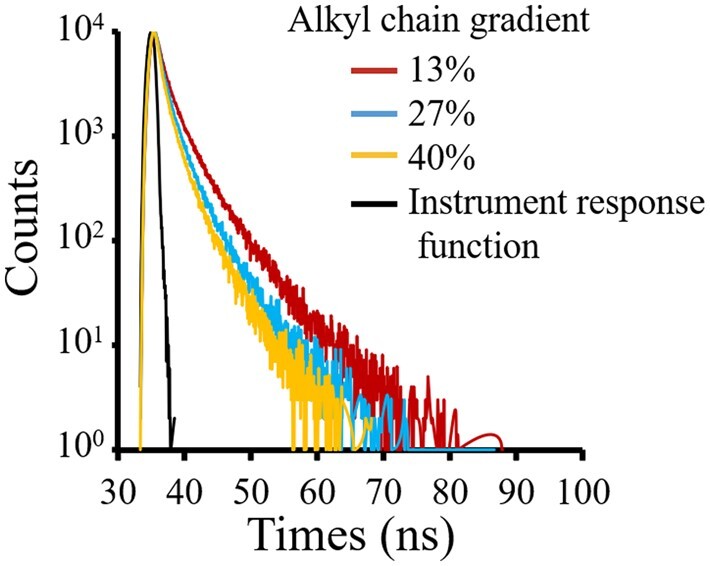
Time-resolved PL decay trace of Alkyl-PEI-LAC-TPE micelles in H_2_O displays a biexponential decay (λ_ex_ = 370 nm, λ_em_ = 480 nm)

To understand if the degree of alkane chain grafting ratios has any effect on the fluorescence property of Alkyl-PEI-LAC-TPE micelles, their fluorescence lifetime was measured. Fluorescence lifetime of fluorescence probes can be used to evaluate the impact of microenvironment (solvent polarity, viscosity, hydrophobicity, pH, etc.) around the fluorophores on their fluorescence property [29]. To obtain fluorescence lifetime, the fluorescence decay curves of gradient alkyl chain Alkyl-PEI-LAC-TPE micelles were measured by transient fluorescence spectrophotometer with the excitation wavelength of 370 nm and the emission wavelength of 480 nm, exhibiting biexponential decay in deionized water system (Fig. 6). The average fluorescence lifetime of Alkyl-PEI-LAC-TPE micelles were 1.71, 1.29 and 1.05 ns according to the alkyl chain ratios of 13%, 27% and 40%, respectively (Table 1). The lower alkane chain grafting ratios, the longer the fluorescence lifetime, and the higher fluorescence intensity of Alkyl-PEI-LAC-TPE micelles.

**Table 1.** The average fluorescence lifetimes of gradient alkyl chain Alkyl-PEI-LAC-TPE micelles (λ_ex_ = 370 nm, λ_em_ = 480 nm)

Grafting rate	Lifetime (τ/ns)		Percentage (%)	Average lifetime (τ/ns)	χ^2^
13%	τ_1_	1.15	0.83	1.71	1.04
	τ_2_	4.39	0.17		
27%	τ_1_	0.92	0.85	1.29	1.02
	τ_2_	3.38	0.15		
40%	τ_1_	0.74	0.86	1.05	1.02
	τ_2_	2.88	0.14		

As previously reported, restriction of intramolecular rotation (RIR) is the main mechanism for the AIE characteristic of TPE fluorophores [6, 30, 31]. The tightly stacked of TPE molecules in the hydrophobic core of Alkyl-PEI-LAC-TPE micelles resulted in the RIR process of TPE and that accounts for its AIE characters. The TPE and alkane chains together formed the hydrophobic core of Alkyl-PEI-LAC-TPE micelles. Therefore, the surrounding alkane chains near TPE molecules may affect the RIR process of TPE, reflecting in the change of fluorescence lifetime and intensity of Alkyl-PEI-LAC-TPE micelles. The lower of alkane chain grafting ratio, the more tightly the stacked of TPE, the higher degree of RIR process, the longer of fluorescence lifetime and the higher of fluorescence intensity of Alkyl-PEI-LAC-TPE micelles.

In addition, the fluorescence lifetime of Alkyl-PEI-LAC-TPE/SPIO nanocomposites have also been studied. As shown in Fig. S4 (online supplementary material), the fluorescence decay curves of Alkyl-PEI-LAC-TPE/SPIO nanocomposites exhibited biexponential decay, the same as Alkyl-PEI-LAC-TPE micelles. And the average fluorescence lifetime of Alkyl-PEI-LAC-TPE/SPIO nanocomposites decreased from 1.53, 1.20 to 1.00 ns as Alkyl-PEI-LAC-TPE grafted with 13%, 27% and 40% alkyl chains, respectively (Table S1, online supplementary material). The fluorescence lifetime of Alkyl-PEI-LAC-TPE/SPIO nanocomposites were slightly shorter than Alkyl-PEI-LAC-TPE micelles. The encapsulated SPIO nanocrystals did not have major impact on the fluorescence properties of Alkyl-PEI-LAC-TPE micelles.

### *T*_2_ relaxivity of Alkyl-PEI-LAC-TPE/SPIO nanocomposites

**Figure 7.**
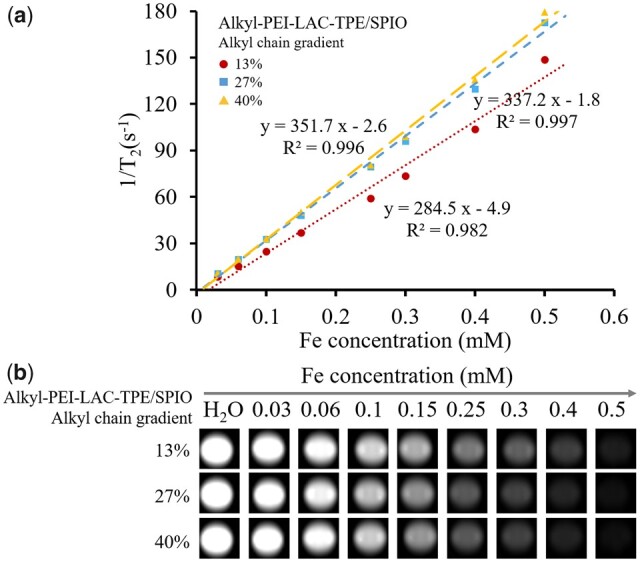
The *T*_2_ relaxation rate (1/*T*_2_, s^−1^) as a function of Fe concentration (mM) for Alkyl-PEI-LAC-TPE/SPIO nanocomposites at 1.5 T. The slope of curve (**a**) is *T*_2_ relaxivity of Alkyl-PEI-LAC-TPE/SPIO; *T*_2_ weighted MRI images (**b**) of Alkyl-PEI-LAC-TPE/SPIO nanocomposites in H_2_O (1.5 T, spin-echo sequence: TR = 5000 ms, TE = 18 ms)

*T*_2_ relaxivities of Alkyl-PEI-LAC-TPE/SPIO nanocomposites with gradient alkyl chains were measured on a 1.5 T clinical MRI scanner (Siemens). IONP is an effective MR *T*_2_ contrast agent, which can significantly shorten the *T*_2_ relaxation time of surrounding water protons [32, 33]. MR images became darker with the increased of iron concentration (Fig. 7b), and the *T*_2_ relaxivity of the Alkyl-PEI-LAC-TPE (13%)/SPIO, Alkyl-PEI-LAC-TPE (27%)/SPIO and Alkyl-PEI-LAC-TPE (40%)/SPIO nanocomposites was 284.6, 337.2 and 351.8 Fe mM^−1^s^−1^, respectively. Results were similar to previous studies that nanocluster containing multiple SPIO nanocrystals performed higher *T*_2_ relaxivities than those containing single SPIO nanocrystal [25]. What is more, the *T*_2_ relaxivities of Alkyl-PEI-LAC-TPE/SPIO nanocomposites became higher with the increased alkane chain grafting ratio. One possible reason might be that there are more SPIO nanocrystals stacked in the hydrophobic core of Alkyl-PEI-LAC-TPE/SPIO nanocomposites with higher alkane chain grafting ratios. Moreover, the *T*_2_ relaxivities of all Alkyl-PEI-LAC-TPE/SPIO nanocomposites were higher than the commercial contrast agent Feridex (120 Fe mM^−1^s^−1^, 1.5 T) [32]. This imaging property indicated that Alkyl-PEI-LAC-TPE/SPIO nanocomposites may be used as an effective *T*_2_ contrast agent or cell labelling probe.

### *In vitro* fluorescence imaging of labelled HeLa cells with Alkyl-PEI-LAC-TPE micelles and Alkyl-PEI-LAC-TPE/SPIO nanocomposites

**Figure 8.**
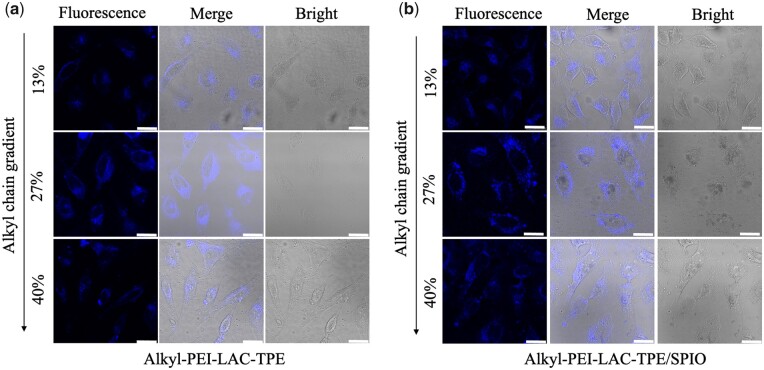
CLSM images of HeLa cells treated with Alkyl-PEI-LAC-TPE (**a**) and Alkyl-PEI-LAC-TPE/SPIO nanocomposites (**b**) at 0.0025 mg TPE/ml (scale bar = 25 µm)

Both Alkyl-PEI-LAC-TPE micelles and Alkyl-PEI-LAC-TPE/SPIO nanocomposites have shown good fluorescence properties, and their efficacies were tested with cell labelling. Alkyl-PEI-LAC-TPE/SPIO nanocomposites shown good biocompatibility to HeLa cells with TPE concentration at 2.5 µg/ml (Fig. S5, online supplementary material). CLSM was used to study the fluorescence imaging of labelled HeLa cells with Alkyl-PEI-LAC-TPE micelles and Alkyl-PEI-LAC-TPE/SPIO nanocomposites. As shown in Fig. 8, after 12 h incubation, Alkyl-PEI-LAC-TPE/SPIO nanocomposites were efficiently internalized by HeLa cells and mainly accumulated in the cytoplasm with high fluorescence intensity, the same as Alkyl-PEI-LAC-TPE micelles. Besides, similar fluorescence intensity in Alkyl-PEI-LAC-TPE micelles and Alkyl-PEI-LAC-TPE/SPIO nanocomposites labelled HeLa cells were detected (Fig. S6, online supplementary material), which further reveal the encapsulated SPIO nanocrystals did not have major impact on the fluorescence properties of Alkyl-PEI-LAC-TPE micelles.

### *In vitro* MRI study of the labelled HeLa cells with Alkyl-PEI-LAC-TPE/SPIO nanocomposites

**Figure 9.**
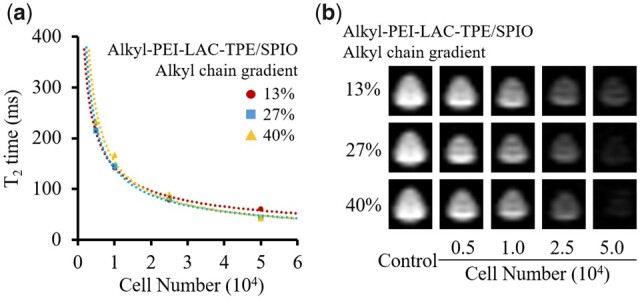
*T*_2_ values of Alkyl-PEI-LAC-TPE/SPIO nanocomposites labelled HeLa cells in microcentrifuge tubes in different cell numbers (**a**); MR images (**b**) of the corresponding samples under SE sequence (3.0 T, TR = 5000 ms, TE = 100 ms)

As a dual-mode MR/fluorescence imaging probe, Alkyl-PEI-LAC-TPE/SPIO nanocomposites should have both MR and fluorescence imaging capabilities. In order to evaluate the probes’ MR visibility, HeLa cells were labelled by Alkyl-PEI-LAC-TPE/SPIO nanocomposites with different Fe concentration (5, 10, 15 and 20 µg/ml) for 12 h. Results (Fig. S7, online supplementary material) showed that Alkyl-PEI-LAC-TPE/SPIO nanocomposites displayed a dose-dependent labelling pattern with excellent labelling efficiency for HeLa cells. MRI study of the labelled HeLa cells with Alkyl-PEI-LAC-TPE/SPIO nanocomposites were evaluated on a 3.0 T clinical MRI scanner. HeLa cells were first labelled with Alkyl-PEI-LAC-TPE/SPIO nanocomposites at an Fe concentration of 5 µg/ml for 12 h. Then, the labelled HeLa cells were collected and homogenously dispersed in gelatine at graded cell numbers. MR images (Fig. 9b) showed that the labelled HeLa cells induced obvious reduction of *T*_2_-weighted signal intensity, which was inversely to the number of cells. As shown in Fig. 9a, *T*_2_ value decreased from 216 to 61 ms with the increasing number of Alkyl-PEI-LAC-TPE (13%)/SPIO labelled HeLa cells, which was similar to the results of Alkyl-PEI-LAC-TPE (27%)/SPIO and Alkyl-PEI-LAC-TPE (40%)/SPIO nanocomposites labelled HeLa cells. These nanocomposites had demonstrated excellent MR imaging sensitivity in label HeLa cells and may find potential applications in cell imaging.

## Conclusion

In conclusion, we have developed an MR/optical imaging probe by encapsulating multiple hydrophobic SPIO nanocrystals in AIE-based amphiphilic Alkyl-PEI-LAC-TPE polymer micelles. Alkyl-PEI-LAC-TPE/SPIO nanocomposites are spherical with controllable size distribution. These nanocomposites exhibited good AIE properties with bright blue emission. Moreover, lower alkylation degree of Alkyl-PEI-LAC-TPE was helpful for longer fluorescence lifetime and higher fluorescence intensity of Alkyl-PEI-LAC-TPE/SPIO nanocomposites. These nanocomposite probes were efficiently internalized by HeLa cells and mainly accumulated in the cytoplasm with high fluorescence intensity. Besides, Alkyl-PEI-LAC-TPE/SPIO nanocomposites had a high *T*_2_ relaxivities, which was higher than that of the commercial MR contrast agent Feridex. The dual-mode imaging probes showed good labelling efficiency for HeLa cells and exhibited excellent MR imaging sensitivity *in vitro* MRI study under clinical MR scanners. In summary, the Alkyl-PEI-LAC-TPE/SPIO nanocomposites as an IONP and AIE molecular-based MR/optical imaging probe may find appropriate applications in biomedical imaging.

## Supplementary Material

rbab023_Supplementary_Data
