# Supplementary material for: Tetraphenylethylene-conjugated polycation covered iron oxide nanoparticles for magnetic resonance/optical dual-mode imaging
Source: Regen Biomater. 2021 Jun 14;8(3):rbab023. doi: 10.1093/rb/rbab023 (PMC8240647; doi:10.1093/rb/rbab023)
Supplement: rbab023_Supplementary_Data [file rbab023_supplementary_data.zip › Supplementary information revised 04-28-2021.doc]

**Tetraphenylethylene-conjugated polycation covered iron oxide nanoparticles for magnetic resonance/optical dual-mode imaging**

Li Yang^1^, Shengxiang Fu^1^, Li Liu^1^, Zhongyuan Cai^1^, Chunchao Xia^2^, Bin, Song^2^, Qiyong Gong^3,4^, Zhiyun Lu^5^ and Hua Ai^1, 2,^ *

^1^National Engineering Research Center for Biomaterials, Sichuan University, Chengdu 610065, P. R. China

^2^Department of Radiology, West China Hospital, Sichuan University, Chengdu 610041, P. R. China

^3^Huaxi MR Research Center (HMRRC), Department of Radiology, West China Hospital of Sichuan University, Chengdu, China

^4^Psychoradiology Research Unit of Chinese Academy of Medical Sciences, Sichuan University, Chengdu, China

^5^Key Laboratory of Green Chemistry and Technology (Ministry of Education), College of Chemistry, Sichuan University, Chengdu 610065, P. R. China

*Correspondence address: National Engineering Research Center for Biomaterials, Sichuan University, Chengdu 610065, P.R. China.

Tel: 86-28-8541-3991

Fax: 86-28-8541-3991

E-mail: huaai@scu.edu.cn

**Figure S1.** The synthesis route of amphiphilic polymer Alkyl-PEI-LAC-TPE.

**Figure S2.** ^1^H NMR spectrum of Alkyl-PEI-LAC-TPE with different grafting degree of alkane chain.

**Figure S3.** Fluorescence images of gradient Alkyl-PEI-LAC-TPE powders under daylight and UV-light.

**Figure S4.** Time-resolved PL decay trace of Alkyl-PEI-LAC-TPE/SPIO nanocomposites with gradient alkyl chain in water displays a biexponential decay. (λ_ex_ = 370 nm, λ_em_ = 480 nm)

**Figure S5.** The cell viability after incubation with Alkyl-PEI-LAC-TPE/SPIO nanocomposites with gradient alkyl chain for 12 h (a) and 24 h (b) at the indicated concentrations were determined using CCK-8, respectively.

**Figure S6.** Mean fluorescence intensity of HeLa cells incubated with Alkyl-PEI-LAC-TPE micelles and Alkyl-PEI-LAC-TPE/SPIO nanocomposites at TPE concentration of 2.5 µg/mL for 12 h.

**Figure S7.** Intracellular iron content of Hela cells after labeled with Alkyl-PEI-LAC-TPE/SPIO nanocomposites. The iron uptake process in Hela cells displays a dose-dependent behavior.

**Table S1.** The fluorescence lifetimes of Alkyl-PEI-LAC-TPE/SPIO nanocomposites with gradient alkyl chain in water (λ_ex_ = 370 nm, λ_em_ = 480 nm)

| Grafting rate | Lifetime (τ/ns) | | Percentage（%） | Averange Lifetime (τ/ns) | χ2 |
| --- | --- | --- | --- | --- | --- |
| 13% | τ_1_ | 1.02 | 0.84 | 1.53 | 1.01 |
|  | τ_2_ | 4.16 | 0.16 |  |  |
| 27% | τ_1_ | 0.86 | 0.86 | 1.20 | 1.05 |
|  | τ_2_ | 3.21 | 0.14 |  |  |
| 40% | τ_1_ | 0.71 | 0.87 | 1.00 | 1.02 |
|  | τ_2_ | 2.93 | 0.13 |  |  |
