# Supplementary material for: Tetraphenylethylene-conjugated polycation covered iron oxide nanoparticles for magnetic resonance/optical dual-mode imaging
Source: Regen Biomater. 2021 Jun 14;8(3):rbab023. doi: 10.1093/rb/rbab023 (PMC8240647; doi:10.1093/rb/rbab023)
Supplement: rbab023_Supplementary_Data [file rbab023_supplementary_data.zip › Response to reviewers 04-28-2021.docx]

Hua Ai, PhD, Professor	*Phone: 86-28-8541-3991*

National Engineering Research Center for Biomaterials	*Fax: 86-28-8541-3991*

Sichuan University, Email：huaai@scu.edu.cn

29 Wangjiang Rd., Chengdu, Sichuan 610064,

P. R. China

April 28, 2021

Dear Prof. Peppas,

We have carefully studied the comments from the reviewers for our work **"Tetraphenylethylene-conjugated polycation covered iron oxide nanoparticles for magnetic resonance/optical dual-mode imaging"** **(ID, RB-2021-047)**. Based on their comments and suggestions, we addressed all the questions and made corresponding changes in the revised manuscript.

***# Reviewer 1:***

Comments to the Author

Recommendation: minor revision.

Comments:

In this article, the authors have developed a MR/optical imaging probe based on Alkyl-PEI-LAC-TPE/SPIO nanocomposites. Amphiphilic polymer Alkyl-PEI-LAC-TPE was designed for encapsulation of hydrophobic SPIO nanocrystals. The modification of lactobic acid on alkyl-PEI was used to reduce the toxicity of PEI and the conjugation of TPE endowed both hydrophobic and AIE features. Alkyl-PEI-LAC-TPE/SPIO nanocomposites can effectively labelled HeLa cells and resulted in high fluorescence intensity and excellent MR imaging sensitivity. As a MR/optical imaging probe, AlkylPEI-LAC-TPE/SPIO nanocomposites may be used in biomedical imaging for certain applications. They performed a complete study of the different grafting degrees of alkane chain (C12) on polymer, all of them were fully characterized and it was performed an exhaustive study. Therefore, I consider that the paper should be published with minor revision:

1. Please adjust the font of Figure 1 and remove the borders for LAC in the spectrum.

**Response:** We thank the reviewer for pointing this out. We have adjusted the font of Figure 1 and removed the borders for LAC in the spectrum.

2. Please adjust the font and size of Figure 6 and S4 and remove the borders for y axis

in Figure 6.

**Response:** We thank the reviewer for pointing this out. We have adjusted the font and size of Figure 6 and S4 and removed the borders for y axis in Figure 6.

***# Reviewer 2:***

Comments to the Author

In this manuscript, the authors report a MRI/Optical dual-mode imaging probe (Alkyl-PEI-LAC-TPE/SPIO) through the Self-assembly of iron oxide nanocrystals (SPIO) and tetraphenylethylene-conjugated alkyl-PEI. And then, the imaging capability were characterized in vitro. The study is well-organized with sufficient proof. Therefore, I would like to recommend publishing this manuscript on Regenerative Biomaterials after minor revisions.

Comments:

(1) In Figure 1, the enlarged 1H NMR spectrum is not clear; please modify it.

**Response:** We thank the reviewer for pointing this out. We have replaced the enlarged ^1^H NMR spectrum in Figure 1 with a clearer picture.

(2) Different alkylation degree of Alkyl-PEI-LAC-TPE were synthesized, please

explain how to control the grafting degree.

**Response:** We thank the reviewer for pointing this out. The synthesis route of Alkyl-PEI-LAC-TPE was displayed in Fig. S1. PEI-LAC was synthesized firstly. Then, TPE-CHO was added for reaction with the amine of PEI-LAC. The LAC and TPE grafting ratios were calculated by elemental analysis according to the changes of ratio of carbon (C) and nitrogen (N) in PEI, PEI-LAC and PEI-LAC-TPE. By controlling the ratio of 1-iodododecane and PEI-LAC-TPE, Alkyl-PEI-LAC-TPE with different alkane grafting degrees were synthesized. In addition, the alkane chain grafting ratio was calculated by the ^1^H NMR of Alkyl-PEI-LAC-TPE according to the integral values at δ 7.22-6.74 ppm and δ 0.81 ppm (Fig. 1 and Fig. S2).

(3) In Figure 5, the figure caption is incomplete, without 5-c and 5-d.

**Response:** We thank the reviewer for pointing this out. We have perfected the figure caption in Figure 5 and highlighted in yellow.

(4) Page 22 Line 52 and Page 24 Line 25; Inconsistent font format, please modify.

**Response:** We thank the reviewer for pointing this out. We have modified the font format in Page 22 Line 52 and Page 24 Line 25 and highlighted in yellow, respectively.

***# Reviewer 3:***

Comments to the Author

The authors designed an aggregation induced emission (AIE) molecule tetraphenylethylene (TPE)-conjugated amphiphilic polyethylenimine (PEI) covered superparamagnetic iron oxide (Alkyl-PEI-LAC-TPE/SPIO nanocomposites), which exhibited good fluorescence property and higher T2 relaxivity in MR/optical dual-mode imaging. Developing MR/optical imaging probes with high sensitivity and high tissue resolution is of great significance. Moreover, the employment of AIE molecule effectively avert aggregation-caused quenching (ACQ) of conventional fluorescence chromophores, resulting in increased fluorescence lifetime. There are some questions needed to be addressed before this manuscript can be accepted for publication in Regenerative Biomaterials.

1. The authors emphasized that reducing alkane chain grafting ratio increased the

fluorescence lifetime of Alkyl-PEI-LAC-TPE/SPIO nanocomposites. Please provide the reasons that the authors discuss gradient alkane chain grafting ratio especially in 13%, 27% and 40%.

**Response:** We thank the reviewer for this comment. As previously reported, restriction of intramolecular rotation (RIR) is the main mechanism for the AIE characteristic of TPE ﬂuorophores. The tightly stacked of TPE molecules in the hydrophobic core of Alkyl-PEI-LAC-TPE micelles resulted in the RIR process of TPE and that accounts for its AIE characters. The TPE and alkane chains together formed the hydrophobic core of Alkyl-PEI-LAC-TPE micelles. The alkane chain may hinder the stack and the RIR process of TPE molecules, resulting in the decrease of fluorescence lifetime and fluorescence intensity of Alkyl-PEI-LAC-TPE micelles. Therefore, the lower of alkane chain grafting ratio, the more tightly the stacked of TPE, the higher degree of RIR process, the longer of fluorescence lifetime, and the higher of fluorescence intensity of Alkyl-PEI-LAC-TPE/SPIO nanocomposites. We have explained the relationship between alkane chain grafting ratio and fluorescence lifetime below Table 1 in the manuscript.

2. In Figure 7b, the cell viability was influenced after incubation of cell with Alkyl-

PEI-LAC-TPE/SPIO nanocomposites for 12 h. Hence the cell viability change should be confirmed after the incubation of cell with gradient nanocomposites. What’s more, the authors should provide the fluorescence intensity change of labeled Hela cells with the increasing of alkyl chain gradient in Figure 7.

**Response:** We thank the reviewer for pointing this out. The cell viability after incubation with Alkyl-PEI-LAC-TPE/SPIO nanocomposites with gradient alkyl chain for 12 h and 24 h at the indicated concentrations were determined using CCK-8, respectively. As shown in the figure below, Alkyl-PEI-LAC-TPE/SPIO nanocomposites show good biocompatibility to HeLa cells after 12 h incubation. At TPE concentration of 25 µg/mL, Alkyl-PEI-LAC-TPE/SPIO nanocomposites with 13% and 27% alkyl chain are toxic to HeLa cells after 24 h incubation, which may due to their more amino groups than Alkyl-PEI-LAC-TPE/SPIO nanocomposites with 40% alkyl chain. In this study, the cell labeling was performed at TPE concentration of 2.5 µg/mL for 12 h, in which concentration, Alkyl-PEI-LAC-TPE/SPIO nanocomposites with gradient alkyl chain show good biocompatibility to HeLa cells.

**Figure S5.** The cells viability after incubation with Alkyl-PEI-LAC-TPE/SPIO nanocomposites with gradient alkyl chain for 12 h (a) and 24 h (b) at the indicated concentrations were determined using CCK-8, respectively.

The mean fluorescence intensity of HeLa cells incubated with Alkyl-PEI-LAC-TPE micelles and Alkyl-PEI-LAC-TPE/SPIO nanocomposites at TPE concentration of 2.5 µg/mL was calculated by Image J (Figure S6). The results showed that the HeLa cells incubation with Alkyl-PEI-LAC-TPE micelles and Alkyl-PEI-LAC-TPE/SPIO nanocomposites displayed similar fluorescence intensity, revealing the encapsulated SPIO nanocrystals did not have major impact on the fluorescence properties of Alkyl-PEI-LAC-TPE micelles. In vitro fluorescence properties evaluation shows that Alkyl-PEI-LAC-TPE micelles with lower alkane chain grafting ratio shows higher fluorescence intensity especially in high TPE concentration (Figure 5). However, there are no significant differences in fluorescence intensity of HeLa cells incubation with Alkyl-PEI-LAC-TPE micelles of gradient alkane chain, which because Alkyl-PEI-LAC-TPE with different alkane chain grafting ratio display similar fluorescence intensity at a low TPE concentration (2.5 µg/mL in Figure. 5, the concentration used in HeLa cells).

**Figure S6.** Mean fluorescence intensity of HeLa cells incubated with Alkyl-PEI-LAC-TPE micelles and Alkyl-PEI-LAC-TPE/SPIO nanocomposites at TPE concentration of 2.5 µg/mL for 12 h.

3. The authors are encouraged to comment on the previous studies, some typical papers were listed herein: Adv. Mater. 2014, 26, 4114–4120; ACS Nano 2017, 11, 9239−9248; ACS Appl. Mater. Interfaces 2014, 6, 10783−10791.

**Response:** We thank the reviewer for pointing this out. We have carefully read the three references listed by the reviewer. Among them, the third reference (ACS Appl. Mater. Interfaces 2014, 6, 10783−10791) which introduces a dual-modal MRI contrast agent with aggregation-induced emission characteristic for liver specific imaging is related to our work. The comment on the third reference has been added in the introduction and highlighted in yellow.
